# Conflict of Medical Evidence as to Personal Injury

**Published:** 1913-06-14

**Authors:** 


### RECENT LEGAL DECISIONS.

### Conflict of Medical Evidence as to Personal Injury.

A farmer, thirty-six years of age, who farmed 300 acres at a rental of £565 and an average yearly profit of £300, was severely injured on the railway, and the jury awarded £4,000 damages. An appeal was taken on the ground that the damages awarded were excessive, but the Court refused to disturb the jury's verdict. The whole question turned on the medical evidence.

In boyhood the pursuer suffered from tuberculous disease of the spine, resulting in curvature in the lower dorsal region and the ossification of three of the vertebræ; but from this he had completely recovered, and become an exceptionally athletic, active, and hard-working man. Unfortunately, the blow which he received in the railway accident involved the spot in the spine where the stiffening from the old ailment existed, and restarted the tubercular condition, and in the course of a very few days it became manifest that his condition was serious and there was a consensus of the doctors on both sides that he must remain on his back for the whole of the year 1913 if he was to be restored to health. The crucial question was: what were the prospects of such restoration?

The points of agreement between the doctors examined were: that the pursuer was an excellent subject, and an excellent patient—a man in good general health, which was well maintained throughout the long confinement to bed, and of equable temper, who bore that confinement with exemplary patience; and there was no symptom of the spinal cord being affected; that what was affected was the bony matter of the vertebræ and the adjoining tissues, in which there was inflammation, causing pressure on, or otherwise affecting, the sensory and not the motor nerves; and that absolute quiescence and open-air treatment for a long continuance, not less than a year, were essential for recovery.

The points of difference between the doctors were that the pursuer's advisers found marked extension of the pain both downward and upward from the seat of the old affection, and from this and other symptoms deduced the conclusion that more vertebræ had been attacked, and consequently, though taking a hopeful, took a more doubtful view of ultimate recovery. While the doctors examined by the defenders were of opinion that the tubercular disease had not spread, and, accounting for the wide area of the pain in other ways, they took a decidedly hopeful view of recovery.

With that narrative before us the following excerpt from the opinion of the Judge who gave the leading opinion is of interest:—

"I have read the medical evidence with great care, and I am glad to say that it has left on my mind the impression that the pursuer's prospect of recovery is hopeful. But its certainty no one can predict. And that is, I think, the situation which the jury had to regard. I think that the position is well illustrated by the evidence of Sir Hector Cameron on behalf of the railway company.

"Asked, near the beginning of his examination in chief, 'Were there any other features of the case from which, when you examined the pursuer in December, you could have been hopeful?' he replied, 'Well, there are no symptoms that made me take a very hopeless view of his case.' Whereas, at the end of his examination in chief, when asked, 'From your examination of the case do you think with good, reasonable treatment and proper rest the case is one which ought to make a good and satisfactory recovery?' he replied, 'That is consistent with all my experience. I do not think there will be any such complications as have been suggested.' Now, I do not attribute to Sir Hector Cameron that, having given a very guarded opinion at first, he was led by the course taken by the examining counsel to throw off his guard and to commit himself to a broad and positive statement. As I read his evidence he will not commit himself at once to a general affirmance of recovery. But having given the data of his diagnosis, he concludes with a favourable prognosis. But it is a guarded opinion after all, and I think the jury were bound to regard the case as one the final result of which no reliable surgeon would predict."

The Court recognised the great difficulty of the question before the jury—the probable effect upon the pursuer's future life—and though some of the Judges thought the award excessive, yet they declined to order a fresh trial.

